# Clinical Characteristics and Outcomes of Surgical Patients with Intensive Care Unit Lengths of Stay of 90 Days and Greater

**DOI:** 10.1155/2017/9852017

**Published:** 2017-07-30

**Authors:** Verena Martini, Ann-Kathrin Lederer, Claudia Laessle, Frank Makowiec, Stefan Utzolino, Stefan Fichtner-Feigl, Lampros Kousoulas

**Affiliations:** Department of General and Visceral Surgery, University of Freiburg Medical Center, Faculty of Medicine, University of Freiburg, Freiburg im Breisgau, Germany

## Abstract

**Background:**

The aim of this study was to evaluate the influence of prolonged length of stay in an intensive care unit (ICU) on the mortality and morbidity of surgical patients.

**Methods:**

We performed a monocentric and retrospective observational study in the surgical critical care unit of the department of surgery at the Medical Center of the University of Freiburg, Germany. Clinical data was collected from patients assigned to the ICU with a length of stay (LOS) of 90 days and greater.

**Results:**

From the total of the 19 patients with ICU LOS over 90 days, ten patients died in the ICU whereas nine patients were discharged to the normal ward. The ICU mortality rate was 52%. The overall survival one year after ICU discharge was 32%. Regarding factors affecting mortality of the patients, significantly higher mortality was associated with age of the patients at the time point of the ICU admission and with postoperative need of renal replacement therapy.

**Conclusions:**

We found a high but in our opinion acceptable mortality rate in surgical patients with ICU LOS of 90 days and greater. We identified age and the need of renal replacement therapy as risk factors for mortality.

## 1. Introduction

Intensive care for patients after major abdominal surgery is nowadays a fixed component of postoperative pathways. The majority of patients after abdominal surgery requires only a few days of intensive care unit (ICU) care, but performing complex operative procedures on older and multimorbid patients has led to an increase in the demand for critical care services and to an increase in the postoperative ICU length of stay (LOS) in individual patients [1–3].

Prolonged ICU stay is not well defined, and the definition depends on the type of ICU and of course on the type of the primary disease of the patient [4–6]. Regarding surgical ICU (SICU) patients, the definition of prolonged ICU LOS ranges between 7 and 21 days [7–9]. Huang et al. [10] found a marked decline of ICU, hospital, and one-year mortality after more than 6 to 16 days of SICU LOS. About 9% of all ICU patients require a maximum of 14 days of ICU care [11], whereas ICU stay longer than 30 days is really uncommon [12, 13].

Major factors that promote prolonged ICU LOS are age of the patient [4], the number of hospital days prior to ICU admission [14], an admission directly from the emergency room, and of course the severity of illness [10, 15].

Prolonged ICU stay is associated with higher mortality rate, longer hospital stay, and a poorer long-term survival [4, 16, 17].

A SICU stay of several days or even a week is not uncommon nowadays, especially in elderly patients with multiple comorbidities undergoing complex abdominal surgery. Rarely, complications warrant an extremely long SICU stay (over 90 days). The goal of this single-center retrospective study was to investigate patient characteristics and outcome in this small group of patients that is, however, consuming a substantial amount of SICU resources.

## 2. Patients and Methods

From January 2005 to December 2015 a total of 12.441 patients were admitted to the surgical intensive care unit of the department of general and visceral surgery at the Medical Center of the University of Freiburg, Germany. Nineteen patients (0.15%) had an ICU LOS of 90 days and greater. The data of these surgical patients were retrospectively analyzed using the electronic patients files, and the survival of the patients was checked with the German residence registration offices and the general practitioners of the patients. Systematic follow-up of all cases was carried out until 31.03.2017.

Patients' demographics, the cardinal diagnosis of the patients, and the indication for the ICU admission are given in Table 1.

The average age of the study population at the time point of ICU admission was 60 and ranged from 26 to 81 years of age. The gender distribution of the patients was 26% females (*n* = 5) and 74% males (*n* = 14).

The median length of ICU stay was 116 days and ranged from 90 to 167 days, whereas the median hospital stay of the patients was 147 days and ranged from 91 to 282 days.

Regarding the indication for the hospital and the ICU admission of the patients, it has to be mentioned that all of them had a surgical principal diagnosis. Eleven of them (58%) had an elective preoperative hospital admission, whereas eight patients (42%) were admitted to the hospital by the emergency room.

The study was approved by the local ethic committee and was performed in accordance with the Declaration of Helsinki.

## 3. Statistical Analysis

For statistical analysis, the SPSS version 23.0 software program was used (SPSS Inc., Chicago, IL, USA). Chi squared test was utilized to test for trends and significance and compare groups of categorical data. *p* < 0.05 was defined as statistically significant. Log rank tests were applied to compare survival times depending on different risk factors. Kaplan-Meier plots were used for illustration.

## 4. Results

Patient characteristics and complications are shown in Tables 2 and 3.

### 4.1. Patient Survival

**Figure 1:**
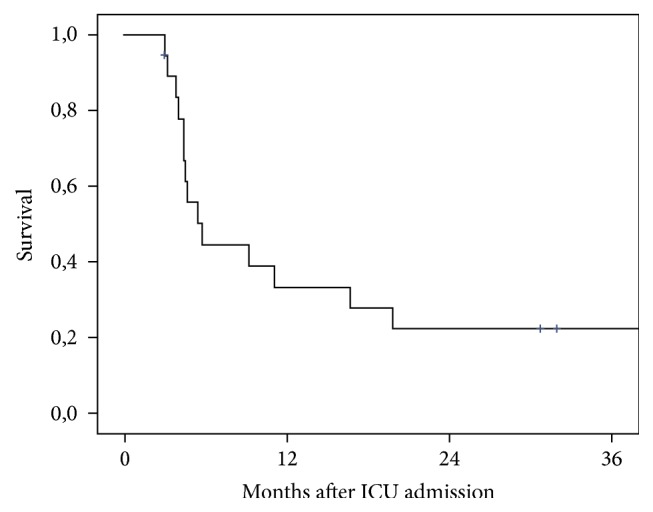
Survival of surgical patient with ICU LOS of 90 days and greater.

The one-year patient survival of our study population was 32% (Figure 1). ICU and hospital mortality rates were both 52%. From the total of the 19 patients with ICU LOS over 90 days, ten patients died in the ICU whereas nine patients were discharged to the normal ward of the hospital where no patient mortality was observed. All of these patients were discharged from our hospital directly to their home, but four of them died at home because of infectious complications (*n* = 2) or because of progress of their neoplastic disease (*n* = 2, one patient with ovarian cancer and one patient with Klatskin Tumor). The average time between discharge from hospital and death of the patient was 230 days, ranging from 36 to 423 days.

**Figure 2:**
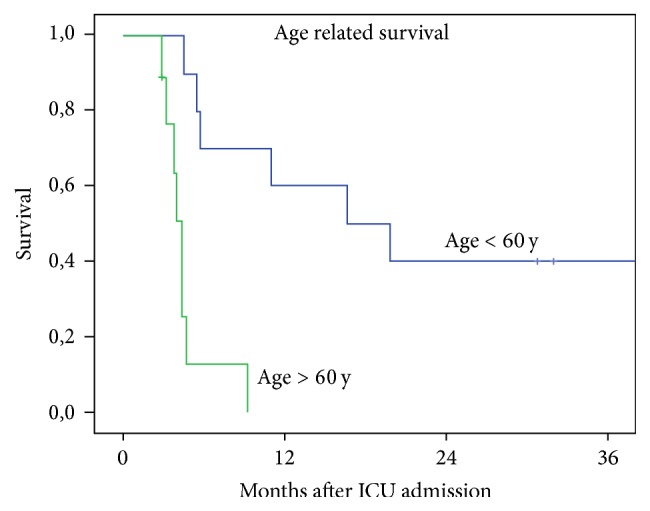
Survival of the patients related to the age at the time point of ICU admission.

**Figure 3:**
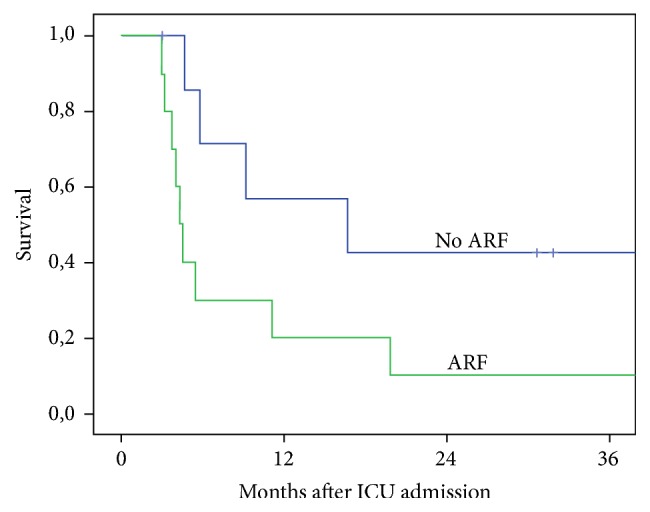
Survival of the patients related to the need of renal replacement therapy (ARF: acute renal failure).

Significantly higher mortality was associated with the age of the patients at the time point of the ICU admission and with the postoperative development of renal failure with need of dialysis. Patients who were older than 60 years of age had a statistically significant higher mortality than patients younger than 60 years of age (88.9% versus 60%; *p* = 0.006) (Figure 2). Moreover, the postoperative development of renal failure with need of hemodialysis was also related to higher ICU mortality rates when compared to patients without need of hemodialysis due to renal failure (86% versus 42%; *p* = 0.006) (Figure 3). Moreover, a trend could be detected for poorer survival of the patients admitted to the ICU as an emergency compared to elective postoperative admissions (63.6% versus 50%), but no statistical significance was identified (*p* = 0.45).

All the other factors tested, such as the number, the severity, and the type of surgical complications, the development of anastomotic leakage or gastrointestinal perforation, the need for and the number of reoperations, the development of postoperative abdominal or pulmonary sepsis, postoperative need for ventilation, the time of ventilation, and the performance of a tracheostomy, were not correlated to higher mortality rates of the surgical patients.

### 4.2. Patient Morbidity and Postoperative Complications

The surgical complications of our study population were stratified according to the modified Clavien classification of postoperative complications [18, 19]. The postoperative course of all patients was highly complicated and led to prolonged ICU LOS, and all patients developed multiple and life-threatening complications. More specifically, eleven patients suffered from gastrointestinal perforation, ten patients had an anastomotic leakage after abdominal surgery, five patients suffered from gastrointestinal ischemia, six patients developed a surgical site infection, and four patients had a postoperative bleeding. The entire patient cohort was in need for multiple reoperations (median of 11 reoperations, ranging from 1 to 41 reoperations).

All patients suffered from an infectious complication. The most frequent kind of infection was occurrence of peritonitis, which was observed in all of the patients. Thirteen patients (68%) were postoperatively diagnosed with pneumonia.

Seventeen patients (89%) received ventilator support (average ventilation time of 835 hours, ranging from 2594 to 16 hours) and in 13 cases (68%) a tracheostomy was performed. Ten patients (52%) suffered postoperatively from acute renal failure and 7 patients (31.5%) were in need for dialysis.

## 5. Discussion

The goal of this retrospective study was to evaluate a small group of patients with extremely long SICU stay (over 90 days), to investigate their characteristics, and to identify risk factors correlating to higher mortality rates. Despite the fact that the performance of complex operative procedures on multimorbid patients has led to an increase of the ICU LOS in individual patients, we know little about the outcome of patients with a very long stay in an intensive care unit. The majority of recent studies focuses on ICU LOS between seven and twenty-one days [7–10] and less information is available on patients who stay in the ICU for over 30 days [20, 21].

Our study presented an ICU and hospital mortality rate of 52%, whereas the one-year patient survival rate was 32%. These outcomes are inferior when compared to the survival rates of trauma patients. As shown by Kisat et al., the ICU mortality rates of trauma patients are high during the first 24 hours on the ICU reaching a value of about 10%. After the first day the mortality rates fall to 3.8% and then steadily increase reaching a maximum of 15% among trauma patients remaining in the intensive care unit from 41 to 90 days [21]. On the other hand, nontrauma patients admitted to a surgical ICU with an ICU LOS of at least 30 days had, compared to trauma patients, inferior outcomes with mortality rates of about 40% [16]. When compared to the nontrauma surgical patients, our study presented similar outcomes for survival of the ICU patients. It could be considered that surgical patients in the ICU, who survive the critical period of the first thirty days, have good outcomes as after this time period the mortality rates of the patients do not become significantly worse. Nevertheless, out of the 19 patients of our study population, nine were discharged to the normal ward of the department and five of them are alive up until today, supporting in this way the concept of never giving up in the critical care medicine. On the other hand, it has to be mentioned that, out of the nine patients discharged from ICU, four of them (44%) died at home in an average period of 230 days after discharge. This is in conclusion to the data presented by Timmers et al. [22] who showed that more than 50% of all surgical patients die within 10 years after discharge from the ICU.

Regarding the factors affecting the mortality of our patients, our study showed that patients who were older than 60 years of age at the time point of the ICU admission had a statistically significant higher mortality comparing to younger patients. This finding correlates with the results of previous studies, as it is demonstrated that the increasing age of the patients is a predictor of prolonged ICU LOS and is associated with increased ICU and hospital mortality [4, 10, 16, 17, 23]. Postoperative acute renal failure with need of hemodialysis was also associated with significant higher mortality rates. 52% of the study population suffered postoperatively from acute renal failure and 32.5% of the patients were in need of dialysis. Friedrich et al. reported that only 16% of the study population required dialysis for acute renal failure [16] but the need of dialysis was clearly identified as predictor of inferior survival rates. Our study demonstrated higher rates of acute kidney injury and acute renal failure, but it has to be assumed that organ failure, such as kidney or lung, is one of the major postoperative problems leading to prolonged ICU LOS in our study population. Regarding these risk factors, it was shown that intensive care patients older than 60 years of age and patients with need of renal replacement therapy had inferior outcomes with a high mortality rate of about 90%. These inferior outcomes are extremely important and should be taken into consideration before taking a decision regarding the continuation of the ICU therapy in critically ill patients.

Our study did not identify any other factors associated with increased ICU, hospital, and overall mortality. As mentioned above all of the surgical patients developed multiple and life-threatening complications, all of them underwent multiple surgical procedures, many developed abdominal or pulmonary sepsis with multiple organ failure, and with two exceptions all of them needed mechanical ventilation. However and in contrast to data presented from previous studies [4, 10, 14–16] none of these factors were in our study associated with higher mortality rates of the patients. These results could be attributed to some potential limitations of our study. It has to be mentioned that the statistical power of our analyses, especially regarding the postoperative complications of the patients, is limited by the sample size. Moreover, wanting to assess survival of patients with ICU LOS of 90 days and greater we had to focus only on these 19 patients and to exclude the rest of the surgical patients, about 12.422, who were treated in the surgical ICU of our department between 2005 and 2015. This selection may be a source of bias resulting in an underestimation of factors affecting the ICU, hospital, and overall mortality of the patients.

To conclude, we found a high but in our opinion acceptable mortality rate in surgical patients with ICU LOS of 90 days and greater. We identified age and the need of renal replacement therapy as risk factors for mortality. Remarkably, there was no further hospital mortality after SICU discharge. Further studies are needed in order to define the outcomes of patients with prolonged ICU LOS, to identify risk factors of higher mortality rates between ICU patients, and of course to help finding the best treatment for patients with very long ICU LOS.

## Figures and Tables

**Table 1:** Patient demographics, cardinal diagnosis, type of hospital and ICU admission, and indication for ICU admission.

Number of patients	19
Gender (male/female) (%)	14/5 (74/26)
Average age at time of ICU admission (years)	60 (26–81)
Median ICU length of stay (days)	116 (90–167)
Median hospital stay (days)	147 (91–282)
Type of hospital admission	
Elective preoperative admission (%)	11 (58)
Emergency admission (%)	8 (42)
Cardinal diagnosis of the patients (%)	
Gastrointestinal cancer	6 (31.6)
Acute pancreatitis	3 (16)
Bleeding/perforation of GI-tract	3 (16)
Ovarian cancer	1 (4.8)
Others	6 (31.6)
Type of ICU admission (%)	
Elective postoperative admission	8 (42)
Emergency admission	11 (58)
Indications for emergency ICU admission (%)	
Pancreatitis	3 (27.3)
Ileus	2 (18.2)
Anastomotic Leak	2 (18.2)
Peritonitis	2 (18.2)
Bleeding	1 (9)
Abdominal compartment	1 (9)

ICU: intensive care unit. GI: gastrointestinal.

**Table 2:** Postoperative complications of surgical patients.

Number of patients	19
Surgical procedures	
Need of reoperation (%)	19 (100%)
Median number of reoperations	11 (1–41)
Type of surgical complications	
GI-perforation	11
Anastomotic Leak	10
Surgical site infection	6
GI ischemia	5
Bleeding	4
Infectious complications (%)	
Abdominal Sepsis	19 (100)
Pulmonary Sepsis	13 (68)
Kidney failure (%)	
Acute renal failure	10 (52,6)
Need of dialysis	7 (36,8)
Respiratory failure	
Need for mechanical ventilation (%)	17 (89)
Median time of mechanical ventilation (hours)	835 (16–2594)
Need of tracheostomy (%)	13 (68)

ICU: intensive care unit. GI: gastrointestinal.

**Table 3:** Characteristics of the 19 surgical patients with intensive care unit lengths of stay of 90 days and greater.

Patient	Type of hospital admission	Cardinal diagnosis	Type of ICU admission	ICU Days	Hospital stay days	Ventilation (hours)	Need for tracheotomy	Need of dialysis	Number of reoperations	Patient status	Patient survival (days)	Death in ICU	Cause of death
1	Elective preoperative admission	Pancreatic Cancer	Elective postoperative admission	167,63	257	1656	Yes	No	23	Dead	176	No	Abdominal sepsis
2	Emergency admission	Perforation of duodenal ulcer	Elective postoperative admission	157,79	282	179	No	No	41	Dead	281	Yes	Pulmonary sepsis
3	Elective preoperative admission	Bladder bleeding after kidney/pancreas transplantation	Emergency, peritonitis after conversion into enteric drainage	149,71	182	321	No	Yes	12	Dead	589	No	Pulmonary sepsis
4	Elective preoperative admission	Choledochus Cyst	Emergency, pancreatitis	135,88	140	0	No	No	8	Living	1007	No	—
5	Emergency	Acute pancreatitis	Emergency, abdominal compartment	134,63	138	1220	Yes	Yes	10	Dead	135	Yes	Abdominal sepsis
6	Elective preoperative admission	Stoma reversal after Hartmann operation	Emergency, anastomotic Leak	129,67	136	2594	Yes	Yes	24	Dead	130	Yes	Abdominal sepsis
7	Elective preoperative admission	Pancreatic cancer	Emergency, bleeding	122,88	136	634	Yes	Yes	13	Dead	134	Yes	Cerebral edema
8	Emergency	Ileus after loop ileostomy	Emergency, ileus	120,88	157	563	Yes	No	16	Living	3750	No	—
9	Elective preoperative admission	Esophageal cancer	Elective postoperative admission	117	121	2518	Yes	No	7	Dead	118	Yes	Abdominal sepsis
10	Emergency	Bleeding of duodenal ulcer	Emergency, peritonitis	114,96	118	588	No	No	13	Dead	115	Yes	Abdominal sepsis
11	Elective preoperative admission	Klatskin Tumor	Elective postoperative admission	114,13	150	0	No	No	1	Dead	507	No	Progress neoplastic disease
12	Elective preoperative admission	Tracheoesophageal Fistula	Elective postoperative admission	105,33	177	62	No	No	1	Dead	162	Yes	Pulmonary Sepsis
13	Emergency	Peritonitis due to anastomotic leak after resection of colon	Emergency, anastomotic Leak	96,04	97	862	Yes	Yes	11	Dead	96	Yes	Abdominal sepsis
14	Emergency	Necrotizing pancreatitis	Emergency, pancreatitis	95,83	118	498	Yes	Yes	3	Living	2865	No	—
15	Emergency	Anastomotic Leak after resection of colon	Elective postoperative admission	93,38	143	16	No	No	8	Dead	141	Yes	Pulmonary sepsis
16	Elective preoperative admission	Ovarian cancer	Emergency, ileus	93,13	130	279	No	No	22	Dead	148	No	Progress neoplastic disease
17	Elective preoperative admission	Esophageal cancer	Elective postoperative admission	91,54	99	1660	Yes	No	5	Living	2055	No	—
18	Elective preoperative admission	Esophageal cancer	Elective postoperative admission	91,21	124	227	No	No	5	Living	210	No	—
19	Emergency	Acute pancreatitis	Emergency, pancreatitis	90,21	91	2003	No	Yes	8	Dead	90	Yes	Pulmonary embolism
